# Risk of cancers of the lung, head and neck in patients hospitalized for alcoholism in Sweden

**DOI:** 10.1054/bjoc.2001.1986

**Published:** 2001-09

**Authors:** P Boffetta, W Ye, H-O Adami, L A Mucci, O Nyrén

**Affiliations:** Unit of Environmental Cancer Epidemiology, International Agency for Research on Cancer, 150 cours Albert-Thomas, Lyon, F-69008, France; Department of Medical Epidemiology, Karolinska Institute, Box 281, Stockholm, S-17177, Sweden; Department of Epidemiology, Harvard School of Public Health, 677 Huntington Avenue, Boston, MA, 02115, USA

**Keywords:** alcohol, laryngeal neoplasms, lung neoplasms, oral cavity neoplasms

## Abstract

Alcoholic patients are at increased risk of cancers of the head and neck but little information is available on the magnitude of the risk for specific sites and for different histological types. We followed 182 667 patients with a hospital discharge diagnosis of alcoholism during 1965–1994, for an average of 10.2 years. We compared their incidence of site- and histological type-specific cancers of the oral cavity, pharynx, larynx and lung with that of the national population. The standardized incidence ratio (SIR) of cancer of the oral cavity and pharynx was 5.33 (95% confidence interval [CI] 5.04–5.64, based on 1207 cases). The SIRs of laryngeal and lung cancer were 4.21 (95% Cl 3.78–4.68, 347 cases) and 2.40 (2.29–2.51, 1880 cases), respectively. The SIR was highest for cancers of the hypopharynx, floor of the mouth, mesopharynx and base of the tongue. The relative excess of lung cancer was similar for squamous cell carcinoma and adenocarcinoma. Low age at first hospitalization was associated with higher SIRs for all sites under study. 25 years after first hospitalization for alcoholism, the cumulative probability of developing a lung cancer was in the order of 5%, for oral and pharyngeal cancer it was 2.5%, and for oesophageal or laryngeal cancer 1% each. Our study shows that the risk of head and neck cancer among heavy drinkers is highest for sites in direct contact with alcohol. The high risk of head and neck neoplasms may justify specific medical attention. © 2001 Cancer Research Campaign http://www.bjcancer.com


BJOC 01-1986 678-682 20/8/01 3:48 pm Page 678

British Journal of Cancer (2001) **85**(5), 678–682
© 2001 Cancer Research Campaign
doi: 10.1054/ bjoc.2001.1986, available online at http://www.idealibrary.com on IDEAL

http://www.bjcancer.com

### Risk of cancers of the lung, head and neck in patients hospitalized for alcoholism in Sweden

**P Boffetta[1,2], W Ye[2], H-O Adami[2,3], LA Mucci[3] and O Nyrén[2]**

[1]Unit of Environmental Cancer Epidemiology, International Agency for Research on Cancer, 150 cours Albert-Thomas, F-69008 Lyon, France; [2]Department of Medical Epidemiology, Karolinska Institute, Box 281, S-17177 Stockholm, Sweden; [3]Department of Epidemiology, Harvard School of Public Health, 677 Huntington Avenue, Boston, MA 02115, USA

**Summary** Alcoholic patients are at increased risk of cancers of the head and neck but little information is available on the magnitude of the risk for specific sites and for different histological types. We followed 182 667 patients with a hospital discharge diagnosis of alcoholism during 1965–1994, for an average of 10.2 years. We compared their incidence of site- and histological type-specific cancers of the oral cavity, pharynx, larynx and lung with that of the national population. The standardized incidence ratio (SIR) of cancer of the oral cavity and pharynx was 5.33 (95% confidence interval [CI] 5.04–5.64, based on 1207 cases). The SIRs of laryngeal and lung cancer were 4.21 (95% CI 3.78–4.68, 347 cases) and 2.40 (2.29–2.51, 1880 cases), respectively. The SIR was highest for cancers of the hypopharynx, floor of the mouth, mesopharynx and base of the tongue. The relative excess of lung cancer was similar for squamous cell carcinoma and adenocarcinoma. Low age at first hospitalization was associated with higher SIRs for all sites under study. 25 years after first hospitalization for alcoholism, the cumulative probability of developing a lung cancer was in the order of 5%, for oral and pharyngeal cancer it was 2.5%, and for oesophageal or laryngeal cancer 1% each. Our study shows that the risk of head and neck cancer among heavy drinkers is highest for sites in direct contact with alcohol. The high risk of head and neck neoplasms may justify specific medical attention. © 2001 Cancer Research Campaign http://www.bjcancer.com

**Keywords**: alcohol; laryngeal neoplasms; lung neoplasms; oral cavity neoplasms

Studies of alcoholic patients have consistently reported increased risks of cancers of the oral cavity and pharynx, oesophagus, liver, larynx, lung and uterine cervix (Sundby, 1967; Pell and D'Alonzo, 1973; Hakulinen et al, 1974; Monson and Lyon, 1975; Robinette et al, 1979; Schmidt and Popham, 1981; Prior, 1988; Adami et al, 1992; Tønnesen et al, 1994; Sigvardsson et al, 1996). Given the correlation between alcohol intake and smoking, it is likely that smoking contributes importantly to the excesses in risk, notably for lung cancer (Blot and Fraumeni, 1996). Relatively small sample sizes have limited the interpretation of previous studies of cancer among alcoholics. For example, in only one study (Tønnesen et al, 1994) were more than 100 cases of head and neck cancer observed. As a consequence, there is limited information on cancer risk by site within head and neck organs and by histological type. Such information would help to better characterize the carcinogenic effect of alcohol, as well as to guide the clinical management of alcoholic patients.

We report the results of the follow-up of a large series of patients hospitalized for alcoholism in Sweden during 1965–1994. Our specific goals were to assess the risk of subsite- and histopathology-specific cancers of the head and neck and lung among this population, and to identify whether this risk is modified by demographic characteristics such as gender and age.

*Received 27 March 2001*
*Revised 8 June 2001*
*Accepted 8 June 2001*

*Correspondence to:* P Boffetta

### METHODS

The study is based on a linkage between the Swedish In-patient Register and the National Cancer Register. We have previously reported details on the In-patient Register (Lagiou et al, 2001).

We considered all records in the In-patient Register with a hospital discharge diagnosis of alcoholism (International Classification of Diseases [ICD]-7 codes 307 and 322 (WHO, 1955); ICD-8 codes 291 and 303 (WHO, 1965); and ICD-9 codes 291, 303 and 305A (WHO, 1975) ) among patients aged 20 or over and hospitalized during 1965–1994. We identified 196 803 unique national registration numbers that met our inclusion criteria. When linking records to the Registries of Population, Death and Emigration, we identified 7790 records with incorrect national registration numbers, 2192 records of patients who died during hospitalization, and 294 records of patients who emigrated before hospitalization; these were all excluded from the study. We finally linked the cohort to the National Cancer Register, which was founded in 1958 and is estimated to be 98% complete (Mattson, 1975), with the aim of identifying cases of cancer that occurred among the patients in the cohort either before (prevalent cases) or after (incidence cases) the first hospital discharge with diagnosis of alcoholism. Cancers were classified according to the ICD-7 (WHO, 1955). We excluded 3405 patients with a prevalent cancer diagnosed before the first hospitalization for alcoholism. A further 455 patients were excluded at various steps of the linkage procedures because of inconsistencies of gender, date of birth or death, etc. We excluded the first year of observation following the first hospitalization in order to reduce selection bias, which could occur if alcoholic patients with a yet undetected subclinical cancer are more likely to be hospitalized than other alcoholic patients. This resulted in the exclusion of 9002 patients who developed a cancer, died or emigrated within 1 year of the first hospital discharge, or were followed up for less than one year. The final cohort of patients with complete data, who were alive and free from cancer 1 year after first hospitalization, comprised 173 665 individuals, of whom 138 195 men and 35 470 women.

The study cohort was thus followed up from the first day of the second year following discharge after the index hospitalization for alcoholism until either occurrence of cancer, death, emigration, or end of follow-up (31 December 1995), whichever occurred first. Incident cancers diagnosed in cohort members excluded latent cancers incidentally detected at autopsy.

We used the standardized incidence ratio (SIR), defined as the ratio of the observed to the expected cases of cancer, as the measure of association. We calculated the expected number of cases by multiplying gender-, 5-year age group- and calendar year-specific cancer incidence rates in Sweden by the observed number of person-years within each stratum of the cohort. We calculated the 95% confidence interval (Cl) for the SIR by the exact method on the assumption of a Poisson distribution of observed cases (Breslow and Day, 1987).

No information was available on severity or duration of alcoholism, or on treatment given. We estimated the SIR of cancer among patients with concurrent alcoholic cirrhosis, alcoholic psychosis or chronic pancreatitis, who might have experienced particularly high intake of alcohol. Additional analyses were based on stratification of patients according to age and year of hospitalization, and time since index hospitalization.

In addition, we calculated the cumulative probability to develop a cancer of interest following the first hospitalization using the Cox regression model, adjusting for age (continuous) and gender. The survival function was estimated by the product-limit method (Kalbfleisch and Prentice, 1980). All analyses were performed using the SAS statistical package, version 6.12 (Sas Institute, 1990).

### RESULTS

Table 1 provides descriptive information on the study cohort. The patients in the cohort provided 1 675 446 person-years of observation, for an average of 10.6 years of follow-up. About one fifth of the patients had their first hospitalization before 1975. Slightly more than half had only one or two hospitalizations for alcoholism, while about one fourth were admitted 6 or more times. The proportion of alcoholic patients who also had a discharge diagnosis of alcoholic cirrhosis was 3% in men and 4% in women.

We observed a total of 1207 cases of oral and pharyngeal cancer, 521 cases of oesophageal cancer, 347 cases of laryngeal cancer and 1880 cases of lung cancer; the numbers of expected cases were 226.3, 94.1, 82.4 and 783.9, respectively. The increase in incidence in the alcoholic cohort was present in both genders and for all 3-digit categories of the ICD-7, with the exclusion of the lip (Table 2). Consistently in men and women, the excess risk was greatest for cancers of the hypopharynx, floor of the mouth, mesopharynx and tongue. In the case of nasopharynx and salivary glands, we found only a modest increase in the number of observed cases over expectation, although the excess in the latter group of neoplasms was statistically significant in men.

Given the large number of oral cancer cases in the cohort, we were able to identify that the excess in lingual cancer was almost completely accounted for by an increase in the incidence of cancers of the base of the organ, while tumours from the mobile (oral) part were not in excess. The excess of cancers of the tonsil, which represent the majority of mesopharyngeal cancers, was similar to other parts of the mesopharynx.

**Table 1** Distribution of patients and person-years by gender and selected characteristics

| | **Men** | | **Women** | |
|---|---|---|---|---|
| | ***n*** | **Person-years** | ***n*** | **Person-years** |
| Age at first hospitalization | | | | |
| <50 | 89 630 | 1 068 278 | 25 050 | 262 518 |
| 50–59 | 25 647 | 260 546 | 5599 | 54 871 |
| 60–69 | 16 069 | 129 060 | 3099 | 25 367 |
| 70+ | 6849 | 38 229 | 1722 | 10 229 |
| Years since first hospitalization | | | | |
| 1–4 | 35 576 | 477 873 | 10 004 | 120 441 |
| 5–9 | 33 912 | 424 957 | 9769 | 101 491 |
| 10–14 | 26 538 | 276 051 | 6733 | 61 371 |
| 15–19 | 25 967 | 145 165 | 6072 | 28 779 |
| 20+ | 16 202 | 33 882 | 2892 | 5436 |
| Year of first hospitalization | | | | |
| 1965–74 | 30 522 | 419 513 | 4227 | 63 285 |
| 1975–84 | 64 672 | 748 599 | 15 261 | 183 805 |
| 1985–94 | 43 001 | 189 816 | 15 982 | 70 428 |
| Number of hospitalizations for alcoholism | | | | |
| 1–2 | 72 542 | 699 591 | 21 814 | 187 601 |
| 3–5 | 27 483 | 289 589 | 6814 | 67 750 |
| 6+ | 38 170 | 368 748 | 6842 | 62 166 |
| Alcoholic cirrhosis | | | | |
| No | 133 566 | 1330 293 | 34 064 | 308 684 |
| Yes | 4629 | 27 635 | 1406 | 8833 |
| Chronic pancreatitis | | | | |
| No | 135 506 | 1 336 902 | 34 999 | 314 115 |
| Yes | 2689 | 21 026 | 471 | 3402 |

© 2001 Cancer Research Campaign 

**Table 2** Standardized incidence ratio of selected neoplasms among alcoholic patients (3-digit ICD7 categories)

| Cancer | | Men | | | Women | | | Both genders | | |
|---|---|---|---|---|---|---|---|---|---|---|
| | ICD-7 code | *n* | SIR | 95% CI | *n* | SIR | 95% CI | *n* | SIR | 95% CI |
| Oral cavity, pharynx | 140 – 148 | 1055 | 5.06 | 4.76 – 5.38 | 152 | 8.45 | 7.16 – 9.91 | 1207 | 5.33 | 5.04 – 5.64 |
| Lip | 140 | 48 | 0.85 | 0.62 – 1.12 | 3 | 1.30 | 0.27 – 3.80 | 51 | 0.86 | 0.64 – 1.14 |
| Tongue | 141 | 215 | 7.40 | 6.44 – 8.46 | 29 | 9.14 | 6.12 – 13.1 | 244 | 7.57 | 6.65 – 8.58 |
| Base of tongue | 141.0 | 59 | 8.25 | 6.28 – 10.6 | 11 | 16.7 | 8.43 – 30.2 | 70 | 8.97 | 6.99 – 11.3 |
| Oral tongue | 141.7 | 26 | 1.12 | 0.73 – 1.65 | 12 | 1.03 | 0.53 – 1.81 | 38 | 1.09 | 0.77 – 1.50 |
| Salivary gland | 142 | 30 | 1.65 | 1.11 – 2.35 | 4 | 1.11 | 0.30 – 2.84 | 34 | 1.56 | 1.08 – 2.18 |
| Floor of the mouth | 143 | 136 | 8.89 | 7.46 – 10.5 | 14 | 14.8 | 8.11 – 24.9 | 150 | 9.24 | 7.82 – 10.8 |
| Other parts of mouth | 144 | 168 | 5.87 | 5.01 – 6.83 | 29 | 7.52 | 5.04 – 10.8 | 197 | 6.06 | 5.25 – 6.97 |
| Mesopharynx | 145 | 191 | 7.85 | 6.78 – 9.05 | 44 | 21.3 | 15.5 – 28.6 | 235 | 8.90 | 7.80 – 10.1 |
| Tonsil[a] | 145.0 | 101 | 7.22 | 5.88 – 8.78 | 23 | 19.3 | 12.2 – 28.9 | 124 | 8.17 | 6.80 – 9.74 |
| Anterior pillar[a] | 145.7 | 2 | 4.65 | 0.56 – 16.8 | 2 | 106 | 12.9 – 383 | 4 | 8.92 | 2.43 – 22.8 |
| Nasopharynx | 146 | 19 | 1.53 | 0.92 – 2.39 | 2 | 2.03 | 0.25 – 7.33 | 21 | 1.56 | 0.97 – 2.39 |
| Hypopharynx | 147 | 235 | 10.5 | 9.20 – 11.9 | 25 | 25.5 | 16.5 – 37.7 | 260 | 11.1 | 9.82 – 12.6 |
| Pharynx unspecified | 148 | 13 | 10.2 | 5.43 – 17.4 | 2 | 29.5 | 3.57 – 106 | 15 | 11.2 | 6.25 – 18.4 |
| Oesophagus | 150 | 465 | 5.26 | 4.79 – 5.76 | 56 | 10.0 | 7.57 – 13.0 | 521 | 5.54 | 5.07 – 6.04 |
| Larynx | 161 | 327 | 4.08 | 3.65 – 4.55 | 20 | 8.91 | 5.44 – 13.8 | 347 | 4.21 | 3.78 – 4.68 |
| Lung | 162 | 1613 | 2.24 | 2.13 – 2.35 | 267 | 4.16 | 3.68 – 4.70 | 1880 | 2.40 | 2.29 – 2.51 |

*n*, number of observed cases; SIR, standardized incidence ratio; CI, confidence interval. [a]The results refer to follow-up during 1965–1992 only.

**Table 3** Standardized incidence ratio of oesophageal and lung cancer among alcoholic patients, by histological type

| Histological type | Men | | | Women | | | Both genders | | |
|---|---|---|---|---|---|---|---|---|---|
| | *n* | SIR | 95% CI | *n* | SIR | 95% CI | *n* | SIR | 95% CI |
| Lung cancer | | | | | | | | | |
| Squamous cell carcinoma | 642 | 2.44 | 2.25 – 2.63 | 63 | 5.30 | 4.07 – 6.78 | 705 | 2.56 | 2.38 – 2.76 |
| Adenocarcinoma | 317 | 2.14 | 1.91 – 2.38 | 76 | 3.25 | 2.56 – 4.07 | 393 | 2.29 | 2.07 – 2.52 |
| Other and unspecified types | 654 | 2.12 | 1.96 – 2.29 | 128 | 4.44 | 3.70 – 5.28 | 782 | 2.32 | 2.16 – 2.49 |
| Small cell carcinoma[a] | 9 | 1.10 | 0.50 – 2.09 | 3 | 1.92 | 0.40 – 5.61 | 12 | 1.23 | 0.64 – 2.15 |
| Oesophageal cancer | | | | | | | | | |
| Squamous cell carcinoma | 397 | 6.39 | 5.78 – 7.05 | 52 | 12.0 | 8.96 – 15.7 | 449 | 6.76 | 6.15 – 7.41 |
| Adenocarcinoma | 26 | 1.45 | 0.95 – 2.12 | 1 | 1.47 | 0.04 – 8.19 | 27 | 1.45 | 0.96 – 2.11 |

*n*, number of observed cases; SIR, standardized incidence ratio; CI, confidence interval. [a]The results on small cell carcinoma refer to follow-up during 1987–1994 only. They are also included in the category 'other and unspecified types of lung cancer'.

**Table 4** Standardized incidence ratio of selected neoplasms among alcoholic patients with diagnosis of alcoholic cirrhosis or chronic pancreatitis

| Cancer | | Alcoholic cirrhosis | | | | Chronic pancreatitis | | | |
|---|---|---|---|---|---|---|---|---|---|
| | ICD-7 code | *n* | SIR | 95% CI | *P* value of difference | *n* | SIR | 95% CI | *P* value of difference |
| Oral cavity, pharynx | 140 – 148 | 84 | 12.5 | 9.9 – 15.4 | < 0.001 | 38 | 11.7 | 8.28 – 16.1 | < 0.001 |
| Tongue | 141 | 13 | 14.0 | 7.47 – 24.0 | 0.04 | 10 | 20.5 | 9.85 – 37.8 | 0.002 |
| Floor of the mouth | 143 | 12 | 25.5 | 13.2 – 44.5 | < 0.001 | 4 | 14.9 | 4.06 – 38.2 | 0.5 |
| Other parts of mouth | 144 | 17 | 17.2 | 10.0 – 27.5 | <0.001 | 6 | 13.1 | 4.81 – 28.5 | 0.1 |
| Oropharynx | 145 | 20 | 26.4 | 16.1 – 40.8 | <0.001 | 6 | 13.8 | 5.06 – 30.0 | 0.4 |
| Hypopharynx | 147 | 18 | 25.3 | 15.0 – 39.9 | <0.001 | 9 | 26.4 | 12.1 – 50.1 | 0.01 |
| Oesophagus | 150 | 18 | 6.12 | 3.63 – 9.67 | 0.8 | 13 | 10.2 | 5.45 – 17.5 | 0.04 |
| Larynx | 161 | 23 | 9.17 | 5.81 – 13.8 | <0.001 | 8 | 6.81 | 2.94 – 13.4 | 0.2 |
| Lung | 162 | 61 | 2.49 | 1.91 – 3.20 | 0.8 | 36 | 3.30 | 2.31 – 4.57 | 0.07 |

*n*, number of observed cases; SIR, standardized incidence ratio; CI, confidence interval.

*© 2001 Cancer Research Campaign*

BJOC 01-1986 678-682 20/8/01 3:48 pm Page 681

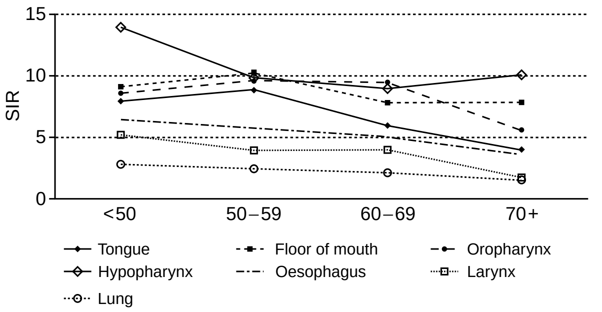

**Figure 1** Standardized incidence ratio (SIR) of selected neoplasms by age at first hospitalization

As expected, the overwhelming majority of cancers from the oral cavity (except salivary glands), the mesopharynx, the hypopharynx and the larynx were classified as squamous cell carcinomas. Notwithstanding, the number of tumours of other histological types was also greater than expected. For example, we observed 5 cases of adenocarcinoma of the tongue vs. 0.3 expected, and one case each of lymphoma of the mesopharynx and of adenocarcinoma of the larynx vs. 0.2 expected for each of them. There was a 2-fold increased risk of squamous cell carcinoma and adenocarcinoma of the lung in the alcoholic cohort (Table 3), while there was little evidence of an important excess of small cell carcinoma, which was first coded as a separate entity in 1987. In the case of oesophageal cancer, an excess risk was noted for squamous cell carcinoma but not for adenocarcinoma (Table 3).

The incidence of oral, pharyngeal, oesophageal or laryngeal cancers did not vary importantly with time since first hospitalization, while for lung cancer the SIR increased with duration of follow-up ($P$ value for trend < 0.01) (detailed data not shown). When patients were stratified according to age at first hospitalization, the excess risk was greater for those who were hospitalized for alcoholism at a young age (Figure 1).

There was a 12-fold excess risk of oral and pharyngeal cancer among alcoholic patients who also had a diagnosis of alcoholic cirrhosis or chronic pancreatitis (Table 4). The greatest increase in risk was observed for cancers of the hypopharynx, mesopharynx and floor of the mouth. There was only a limited overlap between discharge diagnoses of chronic pancreatitis and alcoholic cirrhosis (4% of those patients with either condition).

After 25 years of follow-up, the cumulative risk of oral/pharyngeal cancer is in the order of 3.5% for men aged 50–59 at index hospitalization, and 6% for men aged 60–69. Among women, corresponding risks are in the order of 2% and 4%. The risk of lung cancer is much higher: 7% at 25 years for men aged 50–59 and 13% for those aged 60–69 (in women, corresponding risks are 5% and 10%).

### DISCUSSION

Our study builds upon and expands 2 previous reports on cancer risk among Swedish alcoholics. The first one included 9000 patients hospitalized in Uppsala county during 1965–1983 (Adami et al, 1992), and the second one followed 15 000 women registered in the Temperance Boards between 1917 and 1977 (Sigvardsson et al, 1996). While all subjects included in the Uppsala study were also included – with a longer follow-up – in the present analysis, the overlap with the Temperance Boards study population is unknown. Medical complications of alcoholism favoured inclusion into our cohort, while social and legal complications, sometimes occurring without well-established heavy and chronic alcohol abuse, were the main reason for inclusion in the Temperance Boards cohort.

We found an increased risk of oral, pharyngeal, oesophageal, laryngeal and lung cancer among alcoholics. In general, the increase in risk was greater for digestive than for respiratory organs, in line with those of previous cohort studies of alcoholic patients (Sundby, 1967; Pell and D'Alonzo, 1973; Hakulinen et al, 1974; Monson and Lyon, 1975; Robinette et al, 1979; Schmidt and Popham, 1981; Prior, 1988; Adami et al, 1992; Tønnesen et al, 1994; Sigvardsson et al, 1996). For example, the relative risks (often approximated by SIRs) of oesophageal cancer ranged between 1.9 and 3.2 in studies from the United States and Canada (Monson and Lyon, 1975; Robinette et al, 1979; Schmidt and Popham, 1981), and between 3.8 and 5.5 in studies, including the present one, from the Nordic countries and the United Kingdom (Sundby, 1967; Hakulinen et al, 1974; Prior, 1988; Adami et al, 1992; Tønnesen et al, 1994; Sigvardsson et al, 1996). International differences in the definition of alcoholism and in the entry criteria of the cohort studies might contribute to explain variations in excess risk. Moreover, the general Swedish population, the reference group in our study, had a recorded consumption of 6.3 l of alcohol per person above age 15 in 1991, and an estimated unrecorded consumption (from home-made spirits, imported beverages not declared to customs, etc.) of a further 1.8 l (WHO, 1999), and included 10% of men and 2% of women who were heavy consumers (Kühlorn, 1991). Consumption of tobacco among alcoholics may vary among different study populations.

Advantages of the study include the prospective design and the large size of the cohort which, in combination with a long follow-up, resulted in a uniquely large number of observed cancers. The completeness of the registries ensured essentially complete follow-up, with very little risk of bias due to losses. The histopathological verfication for a high percentage of cancer cases (more than 90% throughout the study) is another strength of our study.

The main innovation of this study was to evaluate the excess risk among alcoholics at specific sites. Most notable increases were observed for cancers of the hypopharynx, the mesopharynx (without appreciable differences between the tonsil and the anterior pillar), the floor of the mouth and the base of the tongue. The increase in risk was intermediate for cancer of other parts of the mouth, which include a miscellaneous group of neoplasms from the alveolus, the internal cheek, the upper gum, and the hard and soft palate, as well as for cancers of the larynx and the oesophagus. The increase in risk was either absent or relatively small – and often non-significant – for cancers of the lip, the oral part of the tongue, the salivary glands, and the nasopharynx. As a whole, these results are consistent with the hypothesis of a carcinogenic effect of alcohol involving direct contact with oral and pharyngeal mucosa, which is also supported by epidemiological studies of moderate alcohol consumption (Tuyns et al, 1988; Boffetta et al, 1992; Franceschi et al, 1992; Kjaerheim et al, 1998). Candidate mechanisms for such a direct effect are DNA binding of acetaldehyde, one of the main metabolites of ethanol and free radicals, as well as reduced DNA repair activity and glutathione trapping by acetaldehyde (Seitz et al, 1998).

Of particular interest are the small but significant increases in the SIRs for cancers of the salivary glands and the nasopharynx

© 2001 Cancer Research Campaign 

BJOC 01-1986 678-682 20/8/01 3:48 pm Page 682

(both SIRs are 1.56). An association between increased alcohol intake and cancer of the salivary glands has been reported in some studies conducted in the general population (Spitz et al, 1990; Muscat and Wynder, 1998). Results on a possible association between alcohol intake and nasopharyngeal cancer are mainly from studies conducted in high-risk areas such as Taiwan and are mostly negative (Chen et al, 1988; Yu and Henderson, 1996). 2 case–control studies from the United States, however, suggested a possible association (Nam et al, 1992; Vaughan et al, 1996).

Despite these advantages, our study suffers from certain limitations. Alcoholism is also associated with additional risk behaviours and hazardous exposures, the average diet of alcoholics being lower in fruits and vegetables than the general population. Most importantly, alcoholics, on average, smoke more than the rest of the population. Our inability to disentangle the effects of smoking from those of alcoholic ingestion is an important limitation of the study. The small increase in risk detected for lung cancer can probably be attributed completely to smoking. A study of alcoholics in Canada found lung cancer risk was 1.7 fold greater than that of the general population, but no excess was found when compared with a population with similar smoking habits (Schmidt and Popham, 1981). However, the ratio of the relative risk of oral and pharyngeal cancer to that of lung cancer (which can be interpreted as 'worst case' adjustment for tobacco smoking, assuming no effect of alcohol on lung cancer and similar relative risk of lung and oral cancers for tobacco smoking) is fairly constant across studies of alcoholics from different parts of the world (range 1.6–3.0; 2.2 in the present study). Moreover, it is not clear that smoking can explain the differences in SIRs within the oral cavity such as the higher risk for the base of the tongue than the oral part.

An additional limitation is the lack of information on the level of alcohol consumption. A plateau in the carcinogenic effect of alcohol at high doses is supported by dose–response analyses conducted in populations with a broad range of exposures (see WCRF/AICR, 1997, for a review). Although we had no information on the time of onset of alcoholism, it seems reasonable to assume that a hospitalization at young age represents early onset of the disease. It also appears biologically plausible that early onset of the disease is associated with a higher cancer risk.

In conclusion, our study clearly demonstrates that alcoholics who require in-hospital care are at substantial risk of head and neck and respiratory cancers. Both alcohol drinking and tobacco smoking are likely to contribute to this excess risk; however, the possible confounding effect of tobacco smoking cannot explain the substantial differences between sites and types of cancer. It turns out that the excesses are greatest for cancers originating from mucous membranes that are in direct contact with alcohol. The possibility of head and neck or respiratory cancer should be kept in mind when caring for these patients.

### REFERENCES

Adami H-O, McLaughlin JK, Hising AW, Wolk A, Ekbom A, Holmberg L and Persson I (1992) Alcoholism and cancer risk: a population-based cohort study. *Cancer Causes Control* **3**: 419–425

Blot WJ and Fraumeni JF Jr (1996) Cancers of the lung and pleura. In: *Cancer Epidemiology and Prevention*, 2nd ed, Schottenfeld D, Fraumeni JF Jr (eds), pp 637–665. Oxford University Press: New York

Boffetta P, Mashberg A, Winkelmann R and Garfinkel L (1992) Carcinogenic effect of tobacco smoking and alcohol drinking on anatomic sites of the oral cavity and oropharynx. *Int J Cancer* **52**: 530–533

Breslow NE and Day NE (1987) Statistical Methods in Cancer Research, Vol II, The Design and Analysis of Cohort Studies (IARC Sci Publ No 82). International Agency for Research on Cancer: Lyon

Chen C-J, Wang Y-F and Shieh T (1988) Multifactorial etiology of nasopharyngeal carcinoma: Epstein-Barr virus, familial tendency and environmental cofactors. In *Head and Neck Oncology Research*, Wolf GT, Carey TE (eds) pp 469–476. Kugler Publications: Amsterdam-Berkeley

Franceschi S, Barra S, La Vecchia C, Bidoli E, Negri E and Talamini R (1992) Risk factors for cancer of the tongue and the mouth: a case-control study from northern Italy. *Cancer* **70**: 2227–2233

Hakulinen T, Lehtimäki L, Lehtonen M and Teppo L (1974) Cancer morbidity among two male cohorts with increased alcohol consumption in Finland. *J Natl Cancer Inst* **52**: 1711–1714

Kalbfleisch JD and Prentice RL (1980) *The Statistical Analysis of Failure Time Data.* John Wiley & Sons, Inc: New York

Kjaerheim K, Gaard M and Andersen A (1998) The role of alcohol, tobacco, and dietary factors in upper aerogastric tract cancers: a prespective study of 10,900 Norwegian men. *Cancer Causes Control* **9**: 99–108

Kühlorn E (1991) Svensk Alkoholpolitik – Facta och Myter. In *Hurmycket tål Svensken?* Atterstam I (ed). Forskingsrådsnamnden: Stockholm

Lagiou P, Ye W, Wedren S, Ekborn A, Nyren O, Trichopoulos D and Adami HO (2001) Incidence of ovarian cancer among alcoholic women: a cohort study in Sweden. *Int J Cancer* **91**: 264–266

Mattson B (1977) The Completeness of Registration in the Swedish Cancer Registry (Stat Rep HS No 15). Stockholm

Monson RR and Lyon JL (1975) Proportional mortality among alcoholics. *Cancer* **36**: 1077–1079

Muscat JE and Wynder EL (1998) A case/control study of risk factors for major salivary gland cancer. *Otolaryngol Head Neck Surg* **118**: 195–198

Nam J-M, McLaughlin JK and Blot WJ (1992) Cigarette smoking, alcohol, and nasopharyngeal carcinoma: a case-control study among U.S. whites. *J Natl Cancer Inst* **84**: 619–622

Pell S and D'Alonzo CA (1973) A five-year mortality study of alcoholics. *J Occup Med* **15**: 120–125

Prior P (1988) Long-term cancer risk in alcoholism. *Alcohol & Alcoholism* **23**: 163–171

Robinette CD, Hrubec Z and Fraumeni JF Jr (1979) Chronic alcoholism and subsequent mortality in World War II veterans. *Am J Epidemiol* **109**: 687–700

Sas Institute (1990) SAS Users Guide, Version 6. Sas Institute, Inc: Cary, NC

Schmidt W and Popham RE (1981) The role of drinking and smoking in mortality from cancer and other causes in male alcoholics. *Cancer* **47**: 1031–1041

Seitz HK, Poschl G and Simanowski UA (1998) Alcohol and cancer. *Recent Dev Alcohol* **14**: 67–95

Sigvardsson S, Hardell L, Przybeck TR and Cloninger R (1996) Increased cancer risk among Swedish female alcoholics. *Epidemiology* **7**: 140–143

Spitz MR, Fueger JJ, Goepfert H and Newell GR (1990) Salivary gland cancer: a case-control investigation of risk factors. *Arch Otolaryngol Head Neck Surg* **116**: 1163–1166

Sundby P (1967) Alcoholism and Mortality (National Institute of Alcohol Research, Publication No. 6). Universitets-forlaget: Oslo

Tønnesen H, Møller H, Andersen JR, Jensen E and Juel K (1994) Cancer morbidity in alcohol abusers. *Br J Cancer* **69**: 327–332

Tuyns AJ, Esteve J, Raymond L, Berrino F, Benhamou E, Blanchet F, Boffetta P, Crosignani P, Del Moral A and Lehmann W (1988) Cancer of the larynx/hypopharynx, tobacco and alcohol: IARC international case-control study in Turin and Varese (Italy), Zaragoza and Navarra (Spain), Geneva (Switzerland) and Calvados (France). *Int J Cancer* **41**: 483–491

Vaughan TL, Shapiro JA, Burt RD, Swanson GM, Berwick M, Lynch CF and Lyon JL (1996) Nasopharyngeal cancer in a low-risk population: defining risk factors by histological type. *Cancer Epidemiol Biomark Prev* **5**: 587–593

WCRF/AICR (1997) Dietary constituents. In: Food, Nutrition and the Prevention of Cancer: a Global Perspective, pp 362–427. American Institute for Cancer Research: Washington

WHO (1955) International Classification of Diseases, 7th rev. (ICD-7). World Health Organization: Geneva

WHO (1965) International Classification of Diseases, 8th rev. (ICD-8). World Health Organization: Geneva

WHO (1975) International Classification of Diseases, 9th rev. (ICD-9). World Health Organization: Geneva

WHO (1999) Global Status Report on Alcohol (WHO/HSC/SAB/99.11). World Health Organization: Geneva

Yu MC and Henderson BE (1996) Nasopharyngeal cancer. In Cancer Epidemiology and Prevention, 2nd ed, Schottenfeld D, Fraumeni JF Jr (eds) pp 603–618. Oxford University Press, New York

 © 2001 Cancer Research Campaign

## References

[PDF_00409] Adami H. O., McLaughlin J. K., Hsing A. W., Wolk A., Ekbom A., Holmberg L., Persson I. (1992). Alcoholism and cancer risk: a population-based cohort study.. Cancer Causes Control.

[PDF_00415] Boffetta P., Mashberg A., Winkelmann R., Garfinkel L. (1992). Carcinogenic effect of tobacco smoking and alcohol drinking on anatomic sites of the oral cavity and oropharynx.. Int J Cancer.

[PDF_00425] Franceschi S., Barra S., La Vecchia C., Bidoli E., Negri E., Talamini R. (1992). Risk factors for cancer of the tongue and the mouth. A case-control study from northern Italy.. Cancer.

[PDF_00428] Hakulinen T., Lehtimäki L., Lehtonen M., Teppo L. (1974). Cancer morbidity among two male cohorts with increased alcohol consumption in Finland.. J Natl Cancer Inst.

[PDF_00433] Kjaerheim K., Gaard M., Andersen A. (1998). The role of alcohol, tobacco, and dietary factors in upper aerogastric tract cancers: a prospective study of 10,900 Norwegian men.. Cancer Causes Control.

[PDF_00438] Lagiou P., Ye W., Wedrén S., Ekbom A., Nyrén O., Trichopoulos D., Adami H. O. (2001). Incidence of ovarian cancer among alcoholic women: a cohort study in Sweden.. Int J Cancer.

[PDF_00445] Muscat J. E., Wynder E. L. (1998). A case/control study of risk factors for major salivary gland cancer.. Otolaryngol Head Neck Surg.

[PDF_00447] Nam J. M., McLaughlin J. K., Blot W. J. (1992). Cigarette smoking, alcohol, and nasopharyngeal carcinoma: a case-control study among U.S. whites.. J Natl Cancer Inst.

[PDF_00450] Pell S., D'Alonzo C. A. (1973). A five-year mortality study of alcoholics.. J Occup Med.

[PDF_00452] Prior P. (1988). Long-term cancer risk in alcoholism.. Alcohol Alcohol.

[PDF_00454] Robinette C. D., Hrubec Z., Fraumeni J. F. (1979). Chronic alcoholism and subsequent mortality in World War II veterans.. Am J Epidemiol.

[PDF_00457] Schmidt W., Popham R. E. (1981). The role of drinking and smoking in mortality from cancer and other causes in male alcoholics.. Cancer.

[PDF_00459] Seitz H. K., Pöschl G., Simanowski U. A. (1998). Alcohol and cancer.. Recent Dev Alcohol.

[PDF_00461] Sigvardsson S., Hardell L., Przybeck T. R., Cloninger R. (1996). Increased cancer risk among Swedish female alcoholics.. Epidemiology.

[PDF_00463] Spitz M. R., Fueger J. J., Goepfert H., Newell G. R. (1990). Salivary gland cancer. A case-control investigation of risk factors.. Arch Otolaryngol Head Neck Surg.

[PDF_00470] Tuyns A. J., Estève J., Raymond L., Berrino F., Benhamou E., Blanchet F., Boffetta P., Crosignani P., del Moral A., Lehmann W. (1988). Cancer of the larynx/hypopharynx, tobacco and alcohol: IARC international case-control study in Turin and Varese (Italy), Zaragoza and Navarra (Spain), Geneva (Switzerland) and Calvados (France).. Int J Cancer.

[PDF_00468] Tønnesen H., Møller H., Andersen J. R., Jensen E., Juel K. (1994). Cancer morbidity in alcohol abusers.. Br J Cancer.

[PDF_00475] Vaughan T. L., Shapiro J. A., Burt R. D., Swanson G. M., Berwick M., Lynch C. F., Lyon J. L. (1996). Nasopharyngeal cancer in a low-risk population: defining risk factors by histological type.. Cancer Epidemiol Biomarkers Prev.

